# Statistical Metamodeling for Revealing Synergistic Antimicrobial Interactions

**DOI:** 10.1371/journal.pone.0015472

**Published:** 2010-11-11

**Authors:** Chia Hsiang Chen, Vincent Gau, Donna D. Zhang, Joseph C. Liao, Fei-Yue Wang, Pak Kin Wong

**Affiliations:** 1 Department of Aerospace and Mechanical Engineering, University of Arizona, Tucson, Arizona, United States of America; 2 GeneFluidics Inc., Monterey Park, California, United States of America; 3 Department of Pharmacology and Toxicology, University of Arizona, Tucson, Arizona, United States of America; 4 Department of Urology, Stanford University, Stanford, California, United States of America; 5 The Key Laboratory for Complex Systems and Intelligence Science, The Institute of Automation, Chinese Academy of Sciences, Beijing, China; 6 Biomedical Engineering and Bio5 Institute, University of Arizona, Tucson, Arizona, United States of America; Charité-University Medicine Berlin, Germany

## Abstract

Many bacterial pathogens are becoming drug resistant faster than we can develop new antimicrobials. To address this threat in public health, a metamodel antimicrobial cocktail optimization (MACO) scheme is demonstrated for rapid screening of potent antibiotic cocktails using uropathogenic clinical isolates as model systems. With the MACO scheme, only 18 parallel trials were required to determine a potent antimicrobial cocktail out of hundreds of possible combinations. In particular, trimethoprim and gentamicin were identified to work synergistically for inhibiting the bacterial growth. Sensitivity analysis indicated gentamicin functions as a synergist for trimethoprim, and reduces its minimum inhibitory concentration for 40-fold. Validation study also confirmed that the trimethoprim-gentamicin synergistic cocktail effectively inhibited the growths of multiple strains of uropathogenic clinical isolates. With its effectiveness and simplicity, the MACO scheme possesses the potential to serve as a generic platform for identifying synergistic antimicrobial cocktails toward management of bacterial infection in the future.

## Introduction

Infectious diseases caused by bacterial pathogens are top causes of health complications and mortality in the world [1]. The pathogens responsible for many human infectious diseases such as urinary tract infection, tuberculosis, gastroenteritis, pneumonia, and wound infections are proven to be highly adept in acquiring mechanisms of antimicrobial resistance [2], [3], [4]. The rapid emergence of multidrug resistant pathogens or “super bugs” is contributed by the widespread injudicious practice of empiric antibiotic usage and infiltration of antibiotics in the food chain, which have accelerated selection and dissemination of resistant pathogens [5]. Consequently, clinicians have fewer treatment options, particularly in the most needy patients. On the other hand, the excessive cost for developing a new antimicrobial and its short useful lifetime render a poor investment returns. Many pharmaceutical companies have de-emphasized their antimicrobial research programs in the past two decades and only few large firms are currently active in building the clinical antibiotic pipeline. As a result, the constant need for new antibiotics has far outpaced the development of new classes of antimicrobials by the pharmaceutical industry (2 in the last 20 years) [5], [6]. This represents a significant threat in public health. Undoubtedly, new sources of antibiotics are highly sought after [7].

The discovery of antibiotics has been traditionally focused on natural products and semi-synthetic tailoring of these natural compounds chemically. Natural substances produced by microorganisms are the most important source of antibiotics. For example, gentamicin is an aminoglycoside antibiotic targeting bacterial ribosomes and is produced by fermentation using *micromonospora*. Many plant-derived materials, such as cinnamon oils and peppermint oils, are also natural antimicrobial agents [8], [9], [10]. In fact, the use of plants with antimicrobial activities for treating wound infections was described in Chinese medicine and other ancient cultures at least 2500 years ago [11]. After the intensive screening of natural antibiotics in the past 50 years, recent antibiotic research effort is migrating to newer sources of natural antimicrobials, such as cyanobacteria and antimicrobial peptides [12], [13]. Synthetic compounds can also provide new avenues in antimicrobials. As an example, sulfamethoxazole is a synthetic antimicrobial that inhibits the folate biosynthetic pathway. Novel libraries of synthetic compounds are being developed to provide improved architectural complexity approaching that of natural products to serve as structural scaffolds for creating new antibiotics [14].

Combination antimicrobial therapy using synergistic antibiotic cocktails, on the other hand, represents an appealing option for treating bacterial infections [7]. A finely tuned mixture of drugs, in many cases, is known to be more effective than monotherapy. A well-known success of drug cocktails in disease treatment is in the fight against human immunodeficiency virus, the virus that causes acquired immune deficiency syndrome [15]. Another example is the treatment of childhood acute lymphoblastic leukemia by the optimization of drug cocktails based on clinical empiricism and trial and error. Evidence for this is seen by the fact that the death rate for acute lymphoblastic leukemia has dropped 90% in the past 25 years [16]. A key advantage of combination therapy using synergistic, or even additive, cocktails is the reduction in drug concentration, which minimizes the toxicity and side effects of many antimicrobials to the host. For instance, high doses of gentamicin can cause renal failure, and permanent losses of equilibrioception and hearing [17], [18]. Combination antimicrobial therapy is, therefore, likely a fruitful option for the treatment of infectious diseases caused by bacterial pathogens. As a matter of fact, co-trimoxazole, a two-drug synergistic combination with trimethoprim and sulphamethoxazole at a 1∶5 ratio, is one of the best selling antibiotics for the treatment of various bacterial infections [19]. Nevertheless, a first hurdle in implementing combination antimicrobial therapy is to identify potent cocktails out of a large number of possible drug combinations. It is difficult to predict synergistic activities between different antimicrobials by studying them individually. Furthermore, the optimal dose of a drug individually could often be different from the most effective dose in a potent cocktail. Identifying potent cocktails out of different concentrations of various antibiotics represent a classical combinatorial optimization problem and the possible combinations increases rapidly with the number of antimicrobials being considered [20], [21]. A systematic procedure for rapidly determining synergistic antimicrobial cocktails is, therefore, necessary to accelerate our progress in combination antimicrobial therapy. In addition, rapid determination of potent antimicrobial cocktails will also improve our ability to promptly response to multidrug resistant pathogens and biological warfare agents in emergency situations and high-risk areas such as temporary clinics established in response to natural and man-made disasters.

Optimization strategies in engineering and computer sciences are well-established approaches for controlling highly complex systems and will likely play an essential role in numerous biomedical sciences and clinical applications [20], [21], [22], [23], [24], [25]. For instance, search algorithms, which iteratively determine the optimal solutions (drug cocktails) in the parametric spaces (different drug combinations), can be applied for determining synergistic drug combinations. A closed-loop optimization strategy was demonstrated for rapid screening of antiviral drug cocktails and identifying cytokine combinations to regulate the NF-κB using signal transduction pathway using a stochastic search algorithm, Gur Game [26]. A Medicinal Algorithmic Combinatorial Screen based on the hill climbing algorithm was demonstrated for screening anti-cancer drug cocktails [27]. A parallel search scheme, Differential Evolution algorithm, was demonstrated for identifying antiviral cocktails for Kaposi's Sarcoma-associated Herpesvirus [28]. These search algorithms, which test drug cocktails sequentially, are especially effective for biological systems with smooth response surfaces in the parametric space. Due to the urgency of many infectious diseases, a metamodel antimicrobial cocktail optimization (MACO) scheme that combines fractional factorial design and stepwise regression is explored in this study. A potential advantage of MACO is that experiment trails are performed in parallel, which dramatically reduces the time required for determining a synergistic cocktail. In general, fractional factorial design is a statistical information-gathering strategy and has been widely applied in engineering research and industrial settings [21], [29]. Instead of brute-force or exhaustive search of all possible drug combinations, which are often impractical in many situations, fractional factorial design can dramatically reduce the number of experiments by defining trials that capture the individual and interactive effects on the response of a complex system. Based on the result, stepwise regression model building procedures can then be performed to extract the potential synergistic interactions between antibiotics.

In this study, the MACO scheme is studied for rapid screening of antimicrobial cocktails from a pool of natural and synthetic antimicrobials. In particular, pathogenic *E. coli* clinical isolates that cause urinary tract infection were chosen as the test organism in this study. A simplified 3-drug search experiment was first performed to illustrate the concept of the scheme for identifying synergistic antimicrobial cocktails. Antimicrobial cocktails were then searched from 5 drugs or 6 drugs to evaluate its robustness in determination of potent drug cocktails. A synergistic cocktail was identified in the study and was evaluated independently using several strains of *E. coli* clinical isolates to evaluate its general applicability. This study will potentially form the foundation of a statistical optimization approach for identifying synergistic antimicrobial cocktails toward the treatment of bacterial infections.

## Results

**Figure 1:**
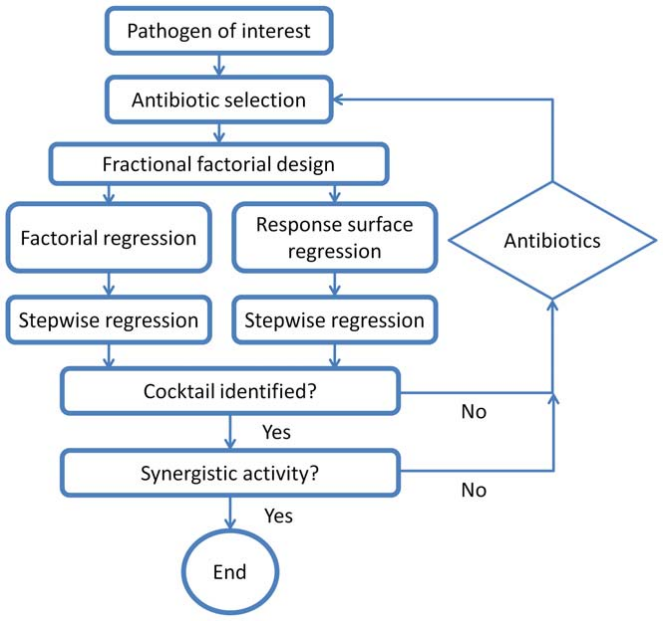
Flowchart representation of the metamodel antimicrobial cocktail optimization (MACO) scheme for identifying potent antimicrobial cocktails. In the MACO scheme, the concentration levels of a set of antibiotics are first chosen based on a pathogen of interest. Fractional factorial design is then performed to select experimental trials that capture the main effects and interactions of antibiotics. Stepwise regression analysis is performed to extract the most effective cocktail in the parametric space. If a synergistic cocktail is identified, the cocktail can be tested for its synergistic activity. If not, the procedure can be repeated with modifications in antibiotics and concentration levels until a synergistic cocktail is identified.

**Figure 2:**
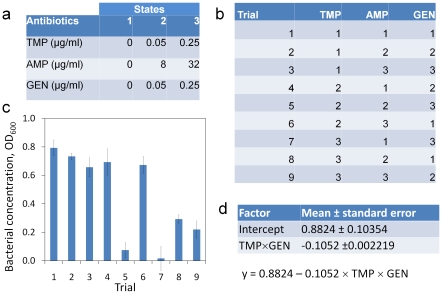
A 3-drug experiment for demonstrating the concept of the MACO scheme. (a) Three concentration levels for each antibiotic are assigned. (b) Fractional factorial experimental design for the three antibiotics considered in this experiment. 9 experimental trials are required. (c) Results of antibiotic susceptibility testing on trials defined in (b). (d) Result of the regression analysis based on the factorial regression and quadratic response surface models. TMP = trimethoprim; AMP = ampicillin; GEN = gentamicin. Data represent absorbance ± standard deviation obtained from 4 independent measurements.

A flowchart representation of the general procedures of the MACO scheme is shown in Figure 1. To illustrate the concept of the MACO scheme, a simplified 3-drug screening experiment was performed using trimethoprim (TMP), ampicillin (AMP), gentamicin (GEN) for inhibiting the growth of an *E. coli* clinical isolate (EC132). Three concentration levels for each antibiotic were assigned (Figure 2a). The lowest concentration considered was zero for all three antibiotics. The highest concentrations for TMP and GEN were chosen to be smaller than their minimum inhibitory concentrations (MIC) since the goal of the experiment is to identify synergistic interaction, instead of individual effects of the antibiotics. EC132 is resistant to AMP and the concentrations of AMP were chosen based on typical values applied in antimicrobial susceptibility testing experiments. Figure 2b shows the fractional factorial design of 9 trials out of the 27 tests required in a full factorial design. The growths of EC132 under different antibiotic combinations were measured (Figure 2c). Inspecting the data revealed that trials 5 and 7, which had relative high concentrations of GEN and TMP, were most effective in inhibiting the bacterial growth. It is intuitive that GEN and TMP may work synergistically for inhibiting bacterial growth. To systematically determine the most potent antimicrobial combination, the data were fed into the statistic software for stepwise regression analyses. The analyses were performed using the fractional regression and quadratic response surface models. In both models, the interaction between TMP and GEN was identified to be the strongest interaction in the antimicrobial combinations. All other factors and interactions were discarded in the forward stepwise regression procedure independent of the model used. The resulting regression model is shown in Figure 2d. In other words, the MACO analysis predicted that the TMP-GEN cocktail had the most potent effect among all individual antibiotics and antibiotic combinations. While the potent cocktail can be determined directly by inspecting the data in this case, the regression analysis in MACO is especially useful for extracting the potent interactions with a larger number of antibiotics.

**Figure 3:**
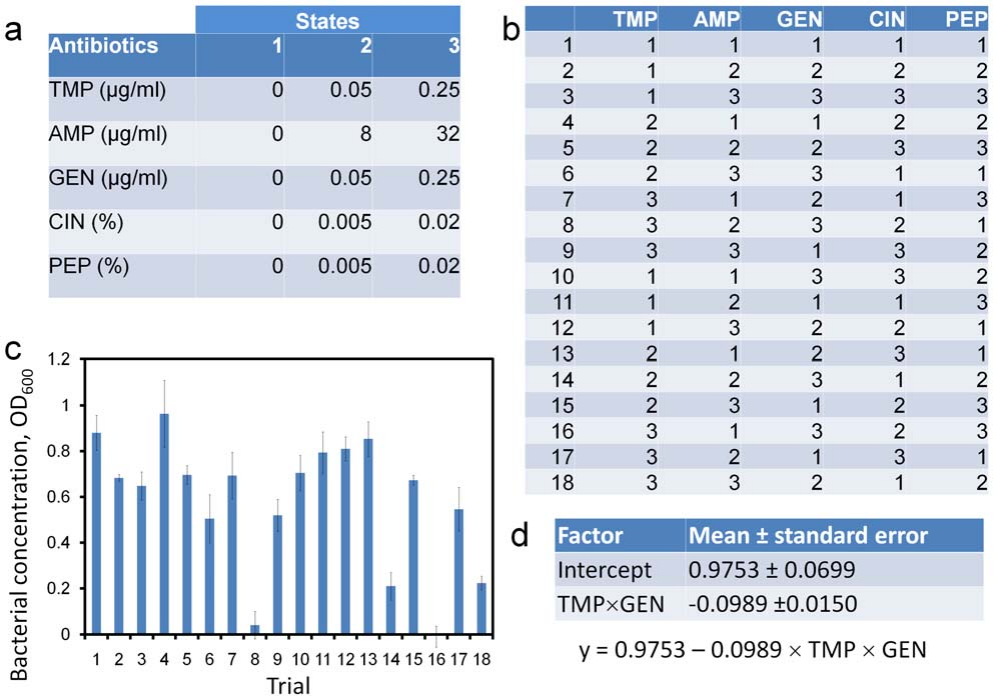
A 5-drug screening experiment using the MACO scheme. (a) Three concentration levels for each antibiotic are assigned. (b) Fractional factorial experimental design for the five antibiotics. 18 experimental trials are required. (c) Bacterial growths with the antibiotic cocktails described in (b). (d) Result of the regression analysis based on the factorial regression and quadratic response surface models. SMX = sulphamethoxazole; CIN = cinnamon oil; PEP = peppermint oil.

**Figure 4:**
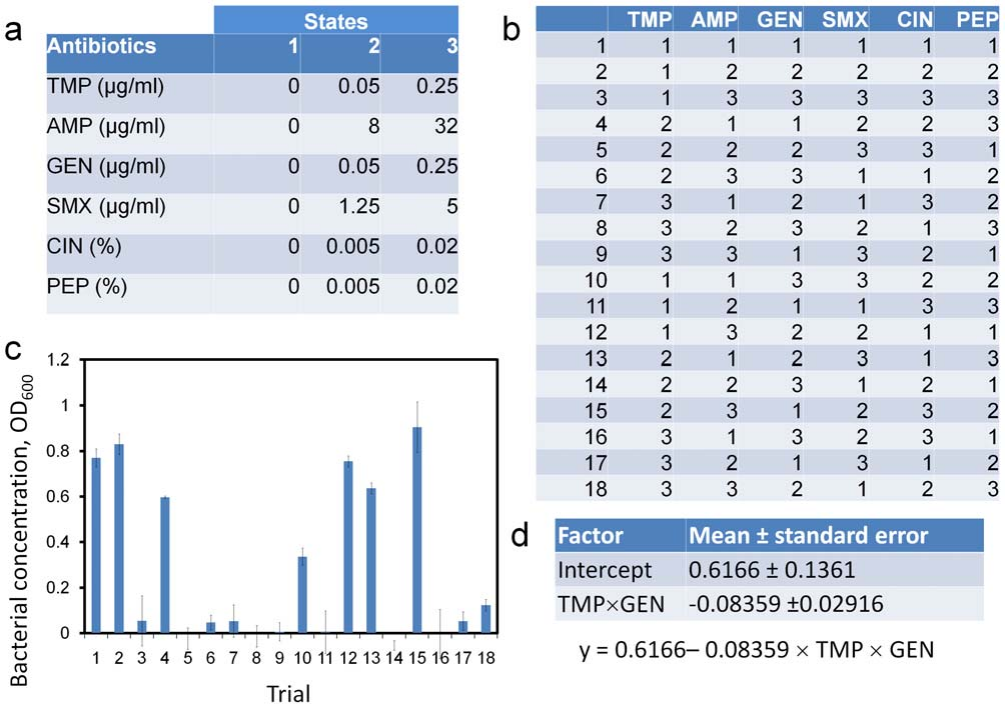
A 6-drug screening experiment. (a) Three concentration levels are assigned for each antibiotic. (b) The 18 experimental trials defined by the fractional factorial design. (c) Experimental results of bacterial growth under different situations. (d) Result of the regression analysis in the MACO scheme.

The MACO scheme was then performed for screening antimicrobial cocktails from a larger number of antibiotics. In addition to TMP, AMP, and GEN, sulfamethoxazole (SMX), cinnamon oil (CIN), and peppermint oil (PEP) were considered in the tests. Similar to the 3-drug experiment, 3 concentrations were assigned for each antibiotic. The highest concentrations of CIN and PEP were chosen to be both 0.02% (Figure 3a and Figure 4a), which was 4-fold smaller than their MIC. EC132 is known to be resistant to SMX and the highest concentration was chosen based on the 1∶19 ratio of the synergistic TMP-SMX combination, which is recommend by the Clinical and Laboratory Standards Institute [30]. The fractional factorial designs for 6 antibiotics required 18 trials out of 729 (3^6^) combinations (Figure 3b and Figure 4b). The bacterial growths under these conditions are shown in Figure 3c and Figure 4c. The results of the regression analyses using the fractional regression and quadratic response surface models are shown in Figure 3d and Figure 4d. In agreement with the 3-drug experiment, TMP and GEN were identified to have the most potent interaction in the 5-drug and 6-durg experiments using both regression models.

In the 5-drug experiment, trials 8 and 16, which had the highest concentrations for both TMP and GEN, were indeed highly effective in inhibiting the growth of the bacteria. The results of the 6-drug experiment were not as intuitive as the other experiments. In the 6-drug experiment, multiple cocktails, trials 5, 8, 9, 11, 14, and 16, were able to effectively inhibit the growth of the pathogens. Based on these data, one could suggest SMX-CIN, TMP-GEN-PEP, CIN-PEP, and TMP-GEN-CIN may processes synergistic activities. A close inspection of the data revealed TMP is critical for inhibiting the bacterial growth based on trials 1, 2, 3 and 10 and TMP-GEN was likely to have the strongest activity based on trials 8 and 16. It should also be note that co-trimoxazole, the combination of TMP and SMX, is also a well-known synergistic cocktail. Interestingly, the regression analyses selected TMP-GEN instead of TMP-SMX as the most potent cocktail for the *E. coli* clinical isolate using both models. It could be a result of the low concentration of TMP in the experiment and the SMX resistant property of EC132. In fact, the concentration of SMX as a synergist was 20-fold higher than the concentration of GEN in the experiment. This suggests TMP may have a stronger interaction with GEN than with SMX.

**Figure 5:**
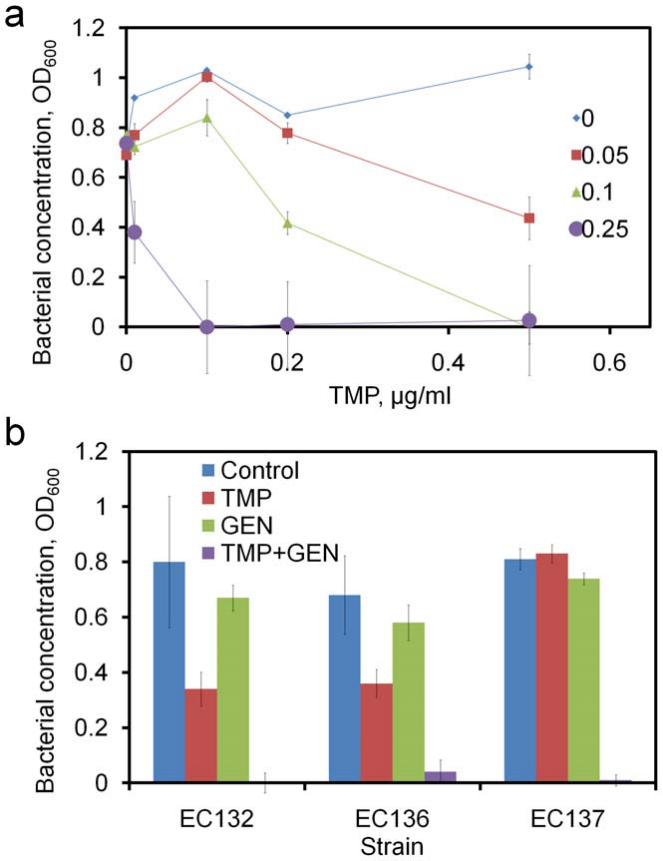
Synergistic activity of TMP and GEN. (a) In the sensitivity analysis of the TMP-GEN cocktails, the dose dependence of TMP on the bacterial growth with different concentration of GEN (in µg/ml) is determined. (b) The antimicrobial cocktail with TMP (0.25 µg/ml) and GEN (0.25 µg/ml) were tested for its individual and interaction effects. The values of EC 132 and EC 137 were not observable in the graph due to the low optical density.

The MACO scheme suggested TMP and GEN interacted synergistically for inhibiting the bacterial growth. A sensitivity analysis was thus performed to evaluate the activity between TMP and GEN (Figure 5a). In the range of 0 to 0.5 µg/ml, TMP showed only a weak-to-moderate effect on the bacterial growth without GEN. It is consistent with the fact that the MIC of TMP for EC132 (i.e., 4 µg/ml) is much higher than the concentration range tested in this experiment (0 to 0.5 µg/ml). When a small amount of GEN is added, TMP displayed dose dependent inhibitions on the bacterial growth. The effect of TMP was significantly enhanced by GEN in a dose dependent manner. For instance, the MIC of GEN could reduce to 0.1 µg/ml with 0.5 µg/ml of TMP and MIC of TMP could reduce to 0.1 µg/ml, a 40-fold reduction, with 0.25 µg/ml of GEN. This supports that TMP and GEN work synergistically and can significantly reduce the MIC of the antibiotics. The synergistic activity of the cocktail was then tested for several strains of uropathogenic *E. coli* clinical isolates (Figure 5b). TMP alone only slightly reduced the bacterial growth for all three strains of *E. coli*. Similarly, GEN alone at 0.25 µg/ml showed a negligible effect on the growth. It should be noted that EC136 and EC137 are sensitivity to GEN at a higher concentration. With the combination of TMP and GEN at the same concentration, total inhibition of the bacteria growth was observed for all three strains of uropathogenic *E. coli*. These results indicate that TMP and GEN exhibit a strong synergistic interaction and that this interaction is applicable to various strains of *E. coli*.

## Discussion

With the MACO scheme that chooses experimental trails selectively and analyzes the data systematically, synergistic antimicrobial cocktails can be quickly determined. Interestingly, the MACO scheme identified the synergistic interaction between TMP and GEN in this study. The mechanistic connection between TMP and GEN at the molecular level is not obvious. TMP inhibits the folate synthesis pathway by interfering the action of bacterial dihydrofolate reductase and GEN inhibits protein synthesis by distorting the structure of the ribosome-RNA complex. This highlights the difficulty of predicting synergistic cocktails by studying them individually and the importance of a systematic approach for identifying synergistic antimicrobial cocktails. The result in this study could provide an insightful starting point for investigating the molecular mechanisms responsible for this synergy [31], [32]. While conventional antibiotic screening focuses on molecular targets one at a time, the MACO approach may lead to a viable pathway for identifying molecules that can be targeted simultaneously for synergistic effects. On the other hand, rapid identification of synergistic cocktails will directly impact the management of bacterial infection. As discussed, high doses of GEN are known to be toxic to the kidney and ears of the host. In fact, GEN typically requires administration by body weight and monitoring of the serum gentamicin level during treatment. Using combination antimicrobial therapy, the MIC of GEN can be reduced for 10-fold with a small amount of TMP. This further supports the appealing nature of combination therapy, which provides effective treatment with minimal toxicity and side effects to the patient.

Optimizing a drug cocktail based on trial and error or clinical empiricism is a challenging task, as evidenced by the time required for developing combination therapies for various diseases. Since the number of possible drug combinations is practically unlimited, a systematic approach for rapidly identifying the most promising cocktails and prioritizing their evaluation is critical. This study demonstrated that potent antimicrobial cocktails could be systematically determined using the MACO scheme. Our result showed the robustness of the procedures of MACO, which consistently identified the synergistic cocktails from different drug combinations. An important advantage of the MACO scheme is the simplicity of the approach that allows the dramatic reduction in the number of experiment trails. The MACO scheme can be easily accessible to researchers with standard microbiology equipments and commercial statistic software. In this study, rapid identification of a potent antimicrobial cocktail was demonstrated from over seven hundred possible combinations in the antimicrobial search space. If the number of antibiotics increases to 13, a full factorial design will require 1,594,323 (3^13^) trials while the fractional factorial design can reduce the number of trial to 27 only.

In addition to identifying novel antimicrobial cocktails for building the next generation antibiotic pipeline in a large-scale pharmaceutical facility, the MACO scheme will also be applicable for clinical management of rapidly spreading in non-traditional settings as a temporary clinic or even in the battlefield. Generally, optimization of complex systems is a fruitful area in engineering, computer science, and operational research. A particular optimization scheme may be uniquely suitable for a specific biomedical problem. For example, search algorithms, such as Gur Game and hill-climbing, were previously demonstrated for searching antiviral and anticancer drugs from large parametric spaces. A major difference between these search algorithms and MACO is that stochastic search algorithms are sequential search methods (i.e., experiments are performed one by one) while all experiment trails in MACO can be operated in parallel. This presents a critical advantage for timely management of rapidly spreading infectious agents in clinical and biodefense settings. The small number of experimental trials in MACO can be easily performed manually or by a laboratory-scale robot at the point of care. In fact, it is possible to perform rapid pathogen identification and antimicrobial susceptibility testing in 2–3 hours in resource limited settings [33], [34], [35], [36] and the MACO analysis can be easily implemented in a laptop computer with commercially available software packages. In summary, the effectiveness and simplicity of MACO renders its potential for rapidly screening synergistic drug cocktails toward the treatment of various diseases in the future.

## Materials and Methods

### Bacterial culture and reagents

Uropathogenic *E. coli* strains EC132, EC136, and EC137 were used as model systems in this study. The pathogens were isolated from clinical urine samples of patients with urinary tract infection as part of a research protocol approved by the Institutional Review Board at Stanford University. Six antibiotics, trimethoprim (TMP), ampicillin (AMP), gentamicin (GEN), sulfamethoxazole (SMX), cinnamon oil (CIN), and peppermint oil (PEP), were considered in this study. EC132 has been tested for its susceptibility to all six antibiotics and is resistant to AMP and SMX [35], [36]. The minimum inhibitory concentrations (MIC) of TMP, GEN, PEP and CIN for EC132 are 4 µg/ml, 1 µg/ml, 0.08% and 0.08% (v/v) respectively. EC137 is sensitive to AMP, GEN, and SMX and EC136 is resistant to AMP. The bacteria colonies were carried on agar plates.

### Antibiotic susceptibility testing (AST)

Before the experiment, the pathogens were cultured in Mueller-Hinton broth in a flask in an orbital shaker at 37°C. After the pathogens were grown to an early exponential phase with an optical density (OD) between 0.2 and 0.6, they were diluted to OD 0.02 with Mueller Hinton broth with the appropriate antimicrobial cocktails, which were freshly prepared before each experiment. The pathogens were then inoculated at 300 rpm and 37°C. The concentrations of bacteria were determined using a microvolume spectrophotometer (Nanodrop 2000). Unless otherwise specified, data represent absorbance ± standard deviation obtained from 4 independent measurements. The quantitative bacterial concentration data were inputed to the statistic program (STATISTICA 9.0, Statsoft Inc) for data analysis. Antimicrobial susceptibility testing was performed using the same procedure to test the synergistic effect of the antimicrobial cocktails. A pathogen was considered to be sensitive to an antimicrobial cocktail if the concentration of the pathogen is less than 10% of the control value in our experiment.

### Fractional factorial design

The MACO scheme is a systematic method for determining potent antimicrobial cocktails. A pathogen of interest, such as a clinical isolate or a biological warfare agent, was first selected. Then, the concentrations (levels) of a set of antibiotics (independent variables) were chosen. To focus on identifying antimicrobial cocktails with synergistic activities, the concentration could be selected to be below the typical minimum inhibitory concentration (MIC) of the antibiotic. In such a situation, the individual effects of antibiotics would unlikely be significant at low concentrations. A fractional factorial design, which selected tests with input parameter values capturing the individual and interactive effects of the drug combinations, was generated using software package. For 3 antibiotics each with 3 concentrations, 9 experimental trials were required out of 27 (3^3^) possible combinations. The reduction in experimental trials increased rapidly with the number of antibiotics being considered. For instance, only 18 trials is required for 6 drugs with 3 concentration levels each out of 729 (3^6^) possible combinations.

### Regression analysis

Experiment trials were performed based on the fractional factorial design in the orthogonal matrix. The experimental data were analyzed using two regression model designs: factorial regression and quadratic response surface regression, since using multiple regressing models could improve the robustness of the analysis. In factorial regression analysis, main factors and interaction effects were both considered. For three drugs (e.g., TMP, AMP, GEN), the coefficients for three main effects (TMP, AMP, and GEN) and four interactions (TMP×AMP, TMP×GEN, AMP×GEN, and TMP×AMP×GEN) were determined. Quadratic response surface regression is similar to factorial regression except that quadratic terms (TMP^2^, AMP^2^, and GEN^2^) were also considered in the analysis. The inclusion of the quadratic response surface regression model will improve the ability of the MACO scheme to capture non-linear effects of the antibiotics. The data were analysis using a forward stepwise regression model-building technique to identify the factors responsible for the synergistic activity. The essences of stepwise regression are to eliminate unnecessary elements and include only factors that have significant effects on the overall response [21]. In this study, a regression analysis is considered to be valid only when both regression models converged to the same result. The stepwise regression analysis was implemented using STATISTICA 9.0 (Statsoft Inc).
