# Correction: Statistical Metamodeling for Revealing Synergistic Antimicrobial Interactions

**DOI:** 10.1371/annotation/d598d976-2604-429b-a76f-14aeca628a8e

**Published:** 2011-07-01

**Authors:** Chia Hsiang Chen, Vincent Gau, Donna D. Zhang, Joseph C. Liao, Fei-Yue Wang, Pak Kin Wong

The first author's name appears incorrectly in the byline, citation, and author contributions. The correct author name is: Hsiang Chia Chen

The correct citation is: Chen HC, Gau V, Zhang DD, Liao JC, Wang F-Y, et al. (2010) Statistical Metamodeling for Revealing Synergistic Antimicrobial Interactions. PLoS ONE 5(11): e15472. doi:10.1371/journal.pone.0015472

The correct author contributions are: Conceived and designed the experiments: HCC VG JCL PKW. Performed the experiments: HCC FYW. Analyzed the data: VG DDZ JCL PKW. Contributed reagents/materials/analysis tools: DDZ JCL PKW. Wrote the paper: HCC PKW. 

